# The Association between Regulatory T Cell Subpopulations and Severe Pneumonia Post Renal Transplantation

**DOI:** 10.1155/2022/8720438

**Published:** 2022-04-09

**Authors:** Quan Zhuang, Haozheng Cai, Min Yang, Bo Peng, Yulin Luo, Ying Zhang, Yingzi Ming

**Affiliations:** ^1^Transplantation Center, The 3rd Xiangya Hospital, Central South University, Changsha 410013, China; ^2^Research Center of National Health Ministry on Transplantation Medicine, Changsha 410013, China

## Abstract

Severe pneumonia accounts for the majority of morbidity and mortality in renal allograft recipients due to immunosuppressant maintenance. Regulatory T cells (Tregs), which are involved in tackling infections under immunosuppressive conditions, are rarely uncovered. We aimed to investigate the relationship between various Treg subpopulations and severe pneumonia after kidney transplantation (KTx). KTx recipients with pneumonia were divided into severe pneumonia and mild pneumonia groups. The frequencies and absolute numbers (Ab No.) of total Tregs (CD4^+^CD25^+^FoxP3^+^), six subsets of Tregs (Helios^+^/^−^, CD39^+/-^, and CD45RA^+^/^−^), and T cells, B cells, and NK cells were assessed from peripheral blood via flow cytometry using the *t* or Mann-Whitney test and receiver operating curve analysis. We also determined the median fluorescence intensity (MFI) of human leukocyte antigen- (HLA-) DR on monocytes and CD64 on neutrophils. Logistic regression was used to identify the risk factors of disease progression, and Pearson's correlation analysis was performed to identify relationships between the measured immune indices and patients' clinical information. Our research indicated that Treg subpopulations were strongly associated with severe pneumonia progression post KTx. Based on the monitoring of Treg subpopulations, better-individualized prevention and therapy might be achieved for patients with severe pneumonia post KTx.

## 1. Introduction

Kidney transplantation (KTx) has been considered as the most effective therapeutic method for end-stage renal disease (ESRD). Although this surgery provides a new lease of life for many ESRD patients, a series of postoperative complications can have a severe effect on patients' survival [1]. Among them, pneumonia is a frequent infectious complication after KTx, which is considered to be a chief cause of morbidity and mortality in renal allograft recipients due to the continued use of immunosuppressants [2, 3].

The long-term application of immunosuppressants has a significant impact on immunity for renal allograft recipients; thus; it is crucial to monitor the immune function of these patients. Recently, regular immune biomarkers have been applied and carried out in clinical practice, including the distribution and absolute counts of CD3^+^, CD4^+^, and CD8^+^ T cells, B cells, natural killer (NK) cells, the CD4/CD8 ratio as well as human leukocyte antigen- (HLA-) DR expression on monocytes, and CD64 expression on neutrophils [4], which have become increasingly useful for diagnosis and treatment in immunosuppressive patients.

Moreover, regulatory T cells (Tregs) are a minor subpopulation (5%–10%) of peripheral T cells (most of which are CD4 positive) [5], and the existence of Tregs is important for self-tolerance. Generally, Tregs are categorized as thymus-derived (natural) and peripheral inducible. In the presence of some anti-inflammatory factors, like transforming growth factor-*β* (TGF-*β*) and interleukin- (IL-) 2, inducible Tregs can be kindled from naïve CD4^+^CD25^−^ cells upon T cell receptor stimulation [6]. Tregs are usually defined as having a high expression of the *α*-subunit of the IL-2 receptor (CD25) and transcription factor FoxP3, as well as a low expression of the IL-7 receptor *α*-subunit (CD127) in both mice and humans [7]. Furthermore, the expression of FoxP3 is necessary for Treg development [8]. Tregs play a crucial role in upholding immune homeostasis and providing protection from autoimmune diseases or allograft rejection [9]. Importantly, KTx patients with chronic rejection have a lower level of peripheral CD4^+^CD25^+^FoxP3^+^ T cells (total Treg) than operationally tolerant and immune-stable patients [10]. *Pneumocystis jirovecii* pneumonia is an opportunistic infection in patients with KTx [11]. Previous studies have shown that in immunocompetent mice with *Pneumocystis* infection, total Tregs are recruited to the lung, and their loss leads to increased pulmonary injury with high levels of T helper (Th) 2 cells and proinflammatory cytokines [12]. However, there are currently no studies elucidating Treg distribution in KTx patients with pneumonia.

Helios (Ikzf2) is one of the important members of the Ikaros transcription factor family, which is detected on 60%–70% of Tregs, and has been proposed as a marker to distinguish natural from peripheral inducible Tregs [13]. Helios can enhance the suppressive capacity of FoxP3^+^ Tregs but not FoxP3^−^ Tregs [14] because Helios upregulates FoxP3 by binding to the FoxP3 promoter [15]. Furthermore, an increased percentage of FoxP3^+^Helios^+^ Treg has been shown to be significantly related to decreased acute graft-versus-host disease in allogeneic bone marrow transplant patients [16]. FoxP3^+^Helios^+^ Tregs were not only correlated to the improvement of graft-versus-host disease but also influenced the prognosis of bacterial pneumonia. A study showed that adoptive transfer of FoxP3^+^Helios^+^ Treg to CBA/Ca mice, prior to *Pneumococcal* infection, prolonged survival and decreased bacterial dissemination from lung to blood [17].

CD39 (ENTPD1) could turn adenosine triphosphate (ATP) to adenosine diphosphate (ADP) and subsequently to adenosine monophosphate (AMP) [18], which is constantly expressed in FoxP3 Tregs in mice. However, CD39 expression in humans is confined to a subset of FoxP3 regulatory effector/memory-like T (TREM) cells [19]. FoxP3^+^CD39^+^ Tregs mainly abrogate the inflammasome-mediated induction of proinflammatory responses by hydrolyzing ATP. Recently, several studies demonstrated that there were remarkably decreased numbers of CD39^+^ Tregs in blood in multiple sclerosis, type 2 diabetes [20, 21]. Compared to wild-type mice, Tregs from CD39-deficient mice could not have enough ability to avert rejection in allogeneic skin grafts [22]. CD39^+^ Tregs were more stable and functional than CD39^−^ Tregs. A previous study has been reported that when adoptively transferring CD39^+^ Tregs to the murine xeno-graft-versus-host model, the survival of mice was longer than that of CD39^−^ Tregs [23]. Furthermore, increased proportions of circulating CD39^+^ Tregs were observed to suppress the replica of human immunodeficiency virus (HIV) and hepatitis B virus (HBV) [24, 25].

In 2006, Hoffmann et al. first defined a naive Treg subpopulation (CD4^+^CD25^+^FoxP3^+^CD45RA^+^) [26], which homogeneously expressed CD62L, CCR7, and CTLA-4 and produced no proinflammatory cytokines. However, under stimulation by alloantigen and IL-12, CD45RA^+^ Treg acquired memory phenotypes, antigen-specific potent suppressive properties, and conversion to an active phenotype (CD25^+^FoxP3^+^CD45RA^−^) [27]. Compared to CD45RA^+^ Tregs, CD45RA^−^ Tregs have robust immunosuppression [28]. Recently, several studies have shown that patients with high levels of CD45RA^−^ Treg have a low rate of transplant rejection [29, 30]. However, elevated CD45RA^−^ Treg may lead to poor prognosis in patients with chronic hepatitis C and HIV infection [31, 32]. Conversely, high levels of CD45RA^+^ Tregs were correlated with low HIV load and replication [31].

Our study was designed to observe the distribution and absolute numbers (Ab No.) of total Treg (CD4^+^CD25^+^FoxP3^+^), six subsets of Tregs (Helios^+/-^, CD39^+/-^, and CD45RA^+/-^), and T cells, B cells, and NK cells (TBNK) between patients with mild and severe pneumonia after KTx. Ours is the first study to investigate different Treg subpopulations in patients with severe pneumonia after KTx and could provide new insights into the monitoring, individualized prevention, and therapy for patients with severe pneumonia post KTx.

## 2. Materials and Methods

### 2.1. Patient Recruitment and Blood Specimen Collection

In this retrospective case-control study, patients diagnosed with pneumonia after KTx were enrolled from June 1, 2019, to October 1, 2020, in our transplant center. Kidney transplant recipients with pneumonia were divided into severe pneumonia (SP) and mild pneumonia (MP) groups. The exclusion criteria included the following: (1) individuals < 18 or >65 years old; (2) other transplant patients (liver, pancreas, bone marrow, etc.); (3) multiorgan transplant recipients; (4) time after transplantation ≤ 3 months post KTx; (5) rejection, tumor, or other site infections; and (6) rituximab treatment. All recipients signed an informed consent form. The study decorum had been assessed and accepted by the Institutional Review Board (Ethics Committee) of the 3rd Xiangya Hospital, Central South University (No. 2018-S347).

Kidney allografts were obtained from donations after circulatory death (DCD) or from close relatives. All surgeries were achieved and were authorized by the DCD Ethics Committee of our hospital. The allocation scheme for the organs was decided by the China Organ Transplant Response System. The induction regimen was made up of antithymocyte globulin (1.00 mg/kg daily for 3 days) or basiliximab (20 mg on days 0 and 4). The maintenance immunosuppressants included calcineurin inhibitors (CNIs), mycophenolate mofetil, and corticosteroids. This regimen was used to maintain a weak immune state and reduce the probability of rejection. Clinical manifestations, meaningful laboratory test results, and prominent radiological examinations were used to diagnose pneumonia patients. Diagnostic criteria of severe pneumonia were based on the American Thoracic Society criteria. The major criteria contained the following: (1) requirement for mechanical ventilation and (2) septic shock. The minor criteria comprised the following: (1) an aspiratory rate > 30 per min, (2) an arterial oxygen pressure/fraction of inspired oxygen ratio < 250, (3) a blood urea nitrogen level > 20 mg/dL, (4) leukopenia < 4 × 10^9^/L, (5) thrombocytopenia, (6) hypothermia, and (7) hypotension requiring aggressive fluid resuscitation. The major criteria ≥ 1 or the minor criteria ≥ 3 [33]. After admission, the patient stopped immunosuppressive treatment. Patients with pneumonia underwent Treg subpopulation tests at the time of pneumonia diagnosis. Most of these patients underwent regular immune monitoring tests. The test was performed at the same time after the patients were admitted to the hospital. Although some patients received multiple tests, only the results of the first test were included. Ethylenediaminetetraacetic acid-K2 anticoagulant tubes were used to collect peripheral blood samples from the patients. Samples were stored and transported at 4°C. Flow cytometry was used to analyze all samples on the day of blood collection.

### 2.2. Regular Immune Status Panel

There are 2 sections in our regular immune status panel. In the first part, BD 6-color TBNK reagent and BD Trucount tubes were used to count the distribution and absolute numbers of leukocytes from peripheral blood. These leukocytes included CD3^+^CD4^+^ T cells, CD3^+^CD8^+^ T cells, NK cells, and CD19^+^ B cells. BD FACSCanto clinical software (BD Biosciences, San Jose, CA, USA) was used to investigate the collected flow results. The median fluorescence intensity (MFI) of HLA-DR on monocytes, and CD64 on neutrophils, was detected by another panel. Fluorochrome-conjugated monoclonal antibodies were used in this study, including anti-CD45-PerCP, anti-CD14-APC-Cy7, anti-HLA-DR-APC, and anti-CD64-PE. Briefly, 50 *μ*L of whole blood was stained with fluorescent antibodies for 15 minutes in the dark (room temperature). The specific steps have been described in the previous study [34]. All samples were analyzed using BD FACSDiva software. BD FACSCanto II was used to perform two panels. Every tube was stopped for testing when 10000 cells were collected.

### 2.3. Treg Subpopulation Test Panel

**Figure 1:**
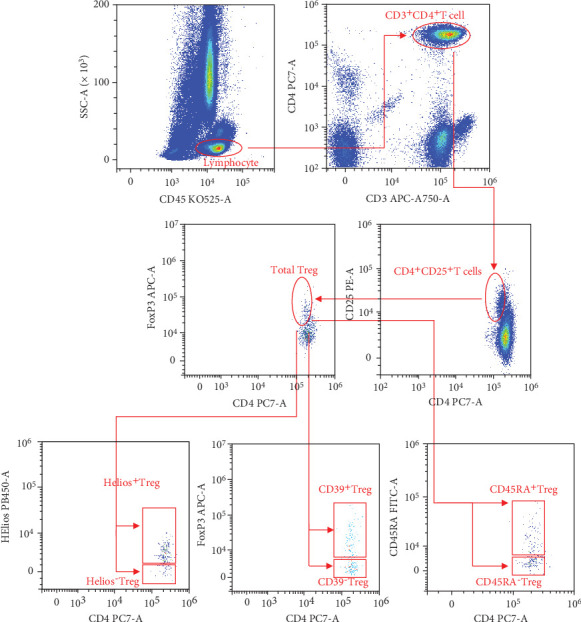
Gating strategies for Treg subpopulations. The first gate was on lymphocytes based on CD45 and SSC-A, and then, CD3 and CD4 were used to gate CD3^+^CD4^+^ T cells. CD25 and FoxP3 together were used to identify total Treg. Helios, CD39, and CD45RA were used to define six subpopulations of Treg: Helios^+/-^ Treg, CD39^+/-^ Treg, and CD45RA^+/-^ Treg.

DuraClone IM Treg tubes were obtained from Beckman Coulter. The details of each fluorochrome-conjugated antibody, the schemes of each fluorochrome channel, and the compensation controls (each of a single color) are shown in Table 1. Briefly, 50 *μ*L of fresh whole blood was added to DuraClone IM Treg Tube 1 and incubated for 15 min (room temperature). Thereafter, phosphate-buffered saline was added to wash the cells. After centrifuging the mixed liquor and aspirating the supernatant, the cell pellet in Tube 1 was resuspended with 50 *μ*L of 100% fetal calf serum. Thereafter, 5 *μ*L of PerFix reagent Buffer 1 was added to Tube 1 and incubated for 15 min in the dark (RT). Subsequently, 400 *μ*L of PerFix nc reagent Buffer 2 (permeabilizing reagent) was added to Tube 1. The contents of DuraClone IM Treg Tube 1 were transferred to Tube 2 and incubated for 60 min in the dark (room temperature). The cells were then rinsed twice and resuspended in 1x PerFix nc Buffer 3. A 13-Color CytoFlex Flow Cytometer (Beckman Coulter, Brea, CA, USA) was used to analyze the samples. The flow cytometer was calibrated using Flow-Set Pro Beads (Beckman Coulter, Brea, CA, USA) daily before use. The Kaluza software (version 1.2; Beckman Coulter, Brea, CA, USA) was carried out to investigate the collected flow cytometric information. We performed compensation by following the AutoSetup Scheduler Application Note “Compensation Setup for High Content DuraClone reagents,” which could be obtained from the Beckman Coulter official weblink. The gating strategies for Treg subpopulations are presented in Figure 1.

### 2.4. Data and Statistical Analysis

Continuous data are presented as the mean ± standard deviation and were compared using the unpaired *t*-test. Variables that do not follow a normal distribution are presented as the median ± interquartile range and were compared using the Mann-Whitney *U* test. Pearson's chi-squared (*χ*^2^) test or Fisher's exact test was used to compare categorical data, where appropriate. The optimal cut-off values of the lymphocytes, CD3^+^CD4^+^ T cells, CD4^+^CD25^+^ T cells, total, Helios^+^, CD39^+^, and CD45RA^−^ Tregs were calculated by applying the receiver operating curve (ROC) analysis. The optimal cut-off values were calculated by “sensitivity + specificity − 1,” and the maximum values were considered as the optimal cut-off values.

Univariate and multivariate logistic regression analyses were applied to screen for risk factors of disease progression. In this section, the indicators which have statistical significance in Tables 2 and 3 were firstly selected to do the univariate logistic analysis. According to the results of the univariate logistic analysis, the variables which have statistical significance were retained to do the next analysis. Then, a forward stepwise multivariate logistic regression analysis was done to select the combination of success factors that have diagnostic value for the occurrence of SP. Improvement of the model was determined from changes in the −2LogLikelihood (−2LL) value. When the model did not improve anymore, no more factors were added. The variables calculated in this part were all numeric variables.

GraphPad Prism 9.0 was used for statistical analysis. Statistical significance was set at *P* < 0.05. Pearson's correlation analysis was performed to identify relationships between the Treg subsets and the clinical information of the patients (with the Pearson *R* > 0.4 (moderate correlation), and *P* < 0.05). The correlation between the results of the regular immune monitoring and Treg subpopulation tests was also calculated and displayed.

## 3. Results

### 3.1. Clinical Characteristics

**Figure 2:**
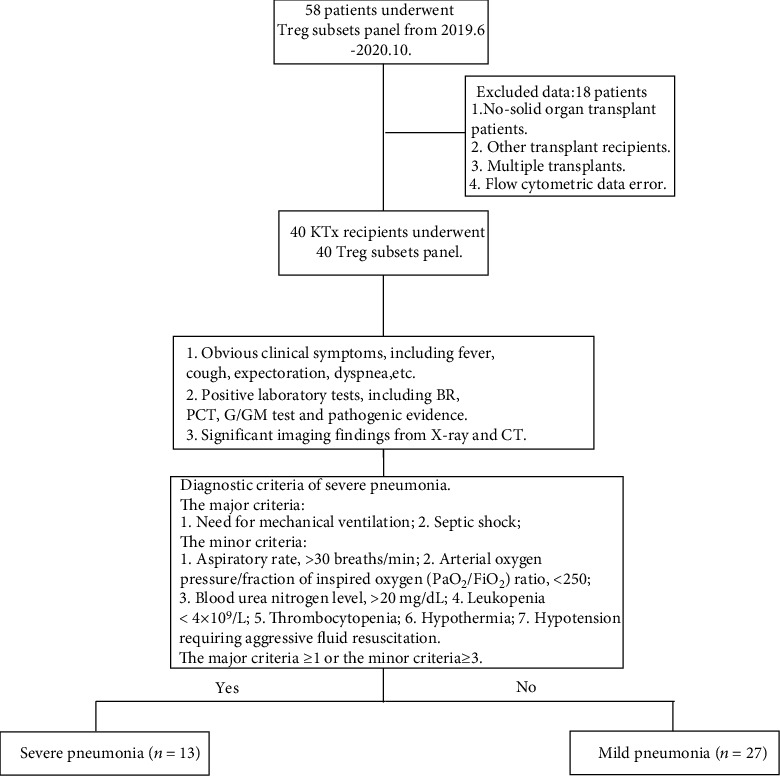
The study flowchart and exclusion criteria. 13 severe pneumonia and 27 mild pneumonia kidney transplant recipients were finally enrolled for analysis.

In total, 27 MP and 13 SP patients were joined. The study flowchart is exhibited in Figure 2. All patients with pneumonia experienced the Treg subpopulation test during the first week after admission. Among these patients, 29 recipients received a regular immune monitoring test at the same time as the Treg test. The clinical characteristics between the MP and SP groups showed no significant difference in the age, sex, body mass index, donor source, time since transplantation, CNI regimen, CRP (C reactive protein), Ca^+^, and white blood cell or platelet counts. The procalcitonin levels were lower in the MP group, compared to the SP group. Patients in the MP group had better allograft function than those in the SP group. Additionally, the creatinine (Cr) and blood urea nitrogen (BUN) levels in the MP group were lower than those in the SP group, and the survival rate in the SP group was 69.23%, which was significantly lower than that in the MP group (100%). The albumin levels in the MP group were higher than those in the SP group. Additionally, the hospital stay (HS) period in the SP group was much longer than that in the MP group. The details of these clinical characteristics are presented in Table 2.

### 3.2. Regular Immune Status

**Figure 3:**
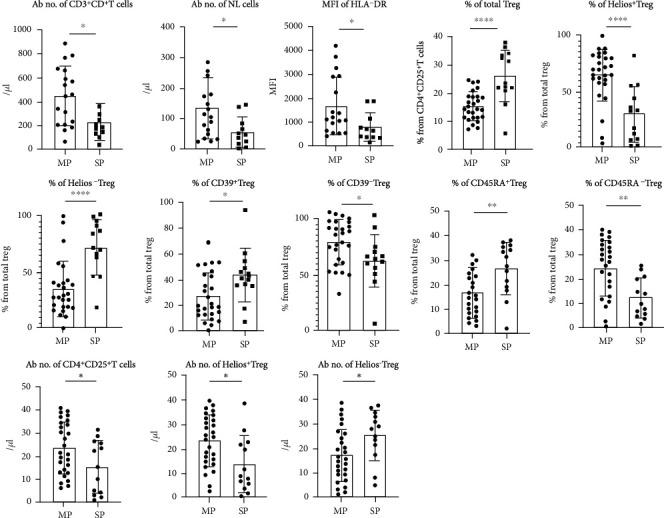
The distribution, Ab No., and MFI of regular immune indexes and Treg subpopulations in MP and SP patients. ∗∗∗∗ means *P* < 0.001, ∗∗ means *P* < 0.05, and ∗ means *P* < 0.1.

Among the 29 patients who underwent regular immune monitoring tests, 18 were MP patients and 11 were SP patients. Compared to the MP group, the SP group was characterized by significantly lower cell counts of CD4^+^ T cells (451.4 ± 58.41/*μ*L vs. 229.2 ± 45.33/*μ*L, *P* = 0.0125) and NK cells (110.50 ± 125.75/*μ*L vs. 45 ± 53.50/*μ*L, *P* = 0.0249). The MFI of HLA-DR on monocytes was significantly lower in the SP group than that in the MP group (1671.00 ± 285.80 vs. 783.60 ± 184.60, *P* = 0.0329) (Table 3) (Figure 3).

### 3.3. Distribution and Counts of Treg Subsets

The Ab No. of CD4^+^CD25^+^ T cells was lower in SP patients compared to MP patients (40.48 ± 97.95/*μ*L vs. 32.56 ± 34.53/*μ*L, *P* = 0.0386). In the SP group, the percentage of total Treg (CD4^+^CD25^+^FoxP3^+^) was significantly higher than that of the MP group (15.26 ± 0.97% vs. 25.94 ± 2.56%, *P* < 0.0001). The expression of Helios in Tregs could be used as a marker to distinguish natural Tregs from inducible Tregs in peripheral blood. The frequency and Ab No. of Helios^+^ Treg in the SP group was significantly lower than that of the MP group (65.09 ± 4.43% vs. 30.25 ± 6.86%, *P* < 0.0001; 4.74 ± 9.93/*μ*L vs. 1.07 ± 3.16/*μ*L, *P* = 0.0127, respectively). Conversely, the percentage and counts of Helios^−^ Treg in the SP group were significantly higher than that in the MP group (33.57 ± 4.47% vs. 69.42 ± 6.58%, *P* < 0.0001; 2.18 ± 2.64/*μ*L vs. 4.73 ± 4.54/*μ*L, *P* = 0.0308, respectively). The percentage of CD39^+^ Tregs in the SP group was significantly higher than that in the MP group (26.74 ± 3.58 vs. 42.95 ± 5.81%, *P* = 0.0177), while the CD39^−^ Treg percentage was significantly lower in the SP group than that in the MP group (71.38% ± 3.48 vs. 56.69 ± 5.81%, *P* = 0.0278). There were similar trends in the frequencies of CD45RA^+^ (11.63 ± 8.09 vs. 27.27 ± 16.14, *P* = 0.0028) and CD45RA^−^ (88.37 ± 8.16 vs. 72.73 ± 15.81, *P* = 0.0018) Tregs. Detailed information is presented in Table 4 and Figure 3.

### 3.4. Correlation Analysis of Treg Subsets with Clinical Information and Regular Immune Status

**Figure 4:**
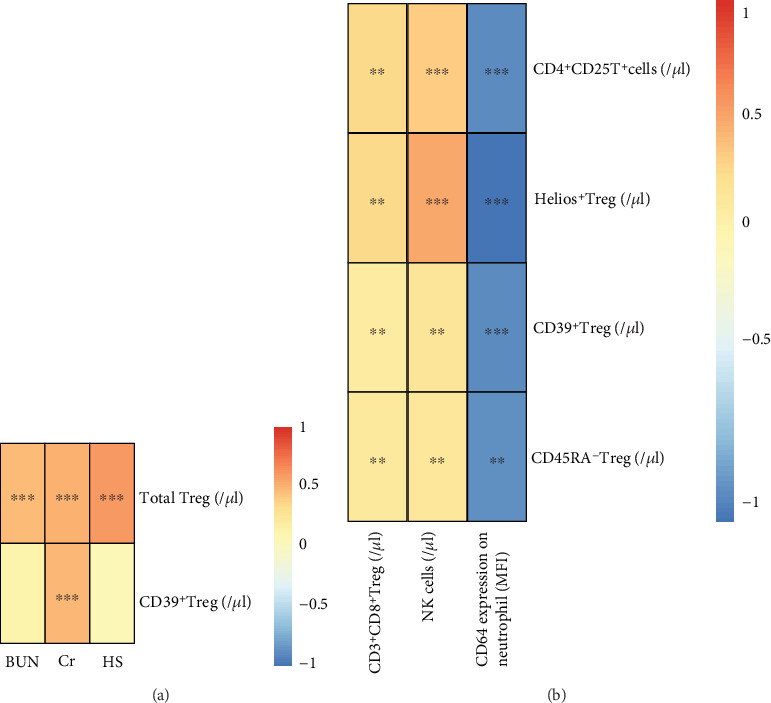
Correlation analysis of Treg subpopulations with clinical information and regular immune status. (a) The heatmap of the correlations between different Treg subpopulations and the clinical information of the patients. (b) The heatmap of the correlations between different Treg subpopulations and the regular immune indexes of the patients. HS: hospital stay period (days); BUN: blood urea nitrogen; Cr: serum creatinine. Red indicates positive correlations while blue indicates negative ones. ∗∗∗ means *P* < 0.01, ∗∗ means *P* < 0.05, and ∗ means *P* < 0.1.

First, we analyzed the correlation between different Treg subpopulations and clinical information (Figure 4(a)). We found that the percentage of total Tregs was positively correlated with the BUN and Cr levels and HS periods (*P* < 0.001) and the CC (correlation coefficient) were 0.47, 0.52, and 0.62, respectively. The Ab No. of CD39^+^ Treg was positively correlated with Cr levels (*P* < 0.01, CC = 0.50). Next, we analyzed the correlation between different Treg subpopulations and regular immune status (Figure 4(b)). We found that the Ab No. of CD3^+^CD8^+^ T cells were positively associated with the absolute counts of CD4^+^CD25^+^ T cells (CC = 0.48, *P* < 0.05) and Helios^+^ (CC = 0.49, *P* < 0.05). The absolute counts of NK cells were also significantly positively correlated with the absolute counts of CD4^+^CD25^+^ T cells (CC = 0.52, *P* < 0.01), Helios^+^ (CC = 0.64, *P* < 0.01), CD39^+^ (CC = 0.42, *P* < 0.05), and CD45RA^−^ Tregs (CC = 0.41, *P* < 0.05). We discovered that the MFI of CD64 on neutrophils was negatively correlated with the absolute counts of CD4^+^CD25^+^ T cells (CC = −0.50, *P* < 0.01), Helios^+^ (CC = −0.60, *P* < 0.01), CD39^+^ (CC = −0.51, *P* < 0.01), and CD45RA^−^ Tregs (CC = −0.49, *P* < 0.05).

### 3.5. ROC and Cut-Off Values of Treg Subsets

**Figure 5:**
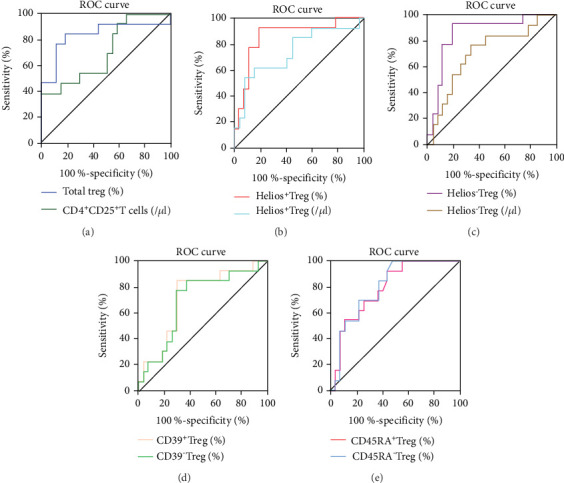
ROC curve of Treg subpopulations in MP and SP patients. (a) ROC curve of CD4^+^CD25^+^ T cells (Ab No.) and total Treg (%). (b) ROC curve of Helios^+^ Treg (% and Ab No.). (c) ROC curve of Helios^−^ Treg (% and Ab No.). (d) ROC curve of CD39^+^ Treg (%) and CD39^−^ Treg (%). (e) ROC curve of CD45RA^+^ Treg (%) and CD45RA^−^ Treg (%).

We selected statistically significant indexes from the results 3.3 to discern patients with SP from MP patients. We analyzed the optimal cut-off values by ROC analysis, and the ROC curves are presented in Figure 5. The area under the curve (AUC) of all indicators was higher than 0.7. The optimal cut-off values were 15.62, 19.87, 56.91, 1.740, 42.06, and 2.835 for CD4^+^CD25^+^ T cells (/*μ*L), total Treg (%), Helios^+^ Treg (%), Helios^+^ Treg (/*μ*L), Helios^−^ Treg (%), and Helios^−^ Treg (/*μ*L), respectively. The optimal cut-off values for CD39^+^ Treg (%), CD39^−^ Treg (%), CD45RA^+^ Treg (%), and CD45RA^−^ Treg (%) were 34.68, 67.84, 12.37, and 87.15, respectively. All AUC and cut-off values are displayed in Table 5.

### 3.6. The Independent Risk Factors

We aimed to foretell the independent risk factors of disease progression based on clinical descriptions and found that the levels of BUN, albumin, Cr, and HS periods were meaningfully related to disease progression by univariate logistic analysis. The multivariate logistic model of clinical information contained BUN and HS. A multivariate logistic analysis was used to show that the BUN (odds ratio (OR) = 1.24, *P* = 0.01) and HS (OR = 1.09, *P* = 0.03) were independent risk factors for disease progression (Table 6). In Table 7, the univariate logistic analysis suggested that the frequencies of total, Helios^+^, Helios^−^ Tregs, CD39^+^, CD39^−^, CD45RA^+^, and CD45RA^−^ Tregs were drastically associated with disease progression. The multivariate logistic model of Tregs contained the following determinants: the percentage of total Tregs and Helios^−^ Tregs. Finally, a multivariate logistic analysis indicated that only the frequencies of total (OR = 1.24, *P* = 0.009) and Helios^−^ (OR = 1.06, *P* = 0.008) Tregs were independent risk factors for disease progression.

## 4. Discussion

This study successfully detailed the distribution and absolute counts of Treg subsets and demonstrated their significance in SP after KTx. We found that the frequencies of total and Helios^−^ Tregs were independent risk factors for severe pneumonia progression after KTx. Therefore, the monitoring of Treg subpopulations and regular immune function may provide a full picture of the immune status for individualized prevention and therapy for severe pneumonia after KTx.

Pneumonia is a major threat to renal allograft recipients [35]. SP can lead to cellular immune disorders and pro- and anti-inflammatory cytokine imbalances [36]. Among the immune cells, NK cells, monocytes, and neutrophils in innate immune responses, as well as CD4^+^/CD8^+^ T cells and B cells in adaptive immune responses, play important roles in defending against the infectious pathogens. In our study, we found the expression of HLA-DR on monocytes and the Ab No. of CD4^+^ T cells and NK cells were lower in SP patients than in MP patients. The decrease of immune cells caused the exacerbation of infection which was contributed to the progression of pneumonia. And the development of the pneumonia eventually led to a significant decrease in graft function. It can even lead to the death of the patients.

Tregs are a small subpopulation of T cells that inhibit effector/memory T cell proliferation and activation and have a dual effect on pneumonia by inhibiting the inflammatory response and promoting tissue repair. In inflammatory diseases, Tregs are involved in maintaining the immune homeostasis mainly by producing anti-inflammatory cytokines, such as IL-10, IL-35, and TGF-*β* [37]. A previous study has demonstrated that long-term use of immunosuppressants increased Tregs in PBMC of patients and mice with organ transplantation [38, 39]. Increased Tregs limited immune activation and prevented the development of graft rejection through secreting anti-inflammatory cytokines and inhibiting the activity of effector T cells [6]. So, patients with high levels of Tregs indicate great graft function and good prognosis. However, in patients with pneumonia, increased Tregs denoted to poor prognosis [40–42]. In our study, we found that the percentage of total Treg was higher in SP patients than in MP patients after KTx. High levels of Tregs prevented T cells from activation, and the maturation and the ability of antigen-presenting cells to activate effector T cells [6], which eventually increased the spread of infection and organ damage. The exacerbation of pneumonia ultimately would lead to decreased graft function in KTx patients [3]. In conclusion, monitoring of Tregs is of great significance for the occurrence and prognosis of infection in patients with KTx. The frequency of total Tregs was an independent risk factor for the development of pneumonia after KTx.

Helios has been reported to stabilize the immunosuppressive function of Tregs, which was also proposed to be a marker for discriminating natural from peripheral inducible Tregs. In our study, we found that the frequency and the Ab No. of Helios^+^ Tregs were lower in SP patients than in MP patients. As the infection worsens, graft function and immune function of patients were gradually declined. Meanwhile, the frequency and Ab No. of Helios^+^ Tregs were also decreased. The increased expression of Helios^+^ Tregs indicated a favorable prognosis in pneumococcal infection and graft function [16, 17]. The decreased expression of Helios^+^ Tregs indicated poor graft function and prognosis in KTx patients with pneumonia. Additionally, we found that the increased frequency of Helios^−^ Tregs was an independent risk factor for the development of pneumonia after KTx. Compared with Helios^+^ Tregs, Helios^−^ Tregs have a reduced suppressive capacity and secrete inflammatory factors [43]. In our research, we demonstrated that the frequency and the Ab No. of Helios^−^ Tregs were higher in SP patients than in MP patients. The increased infiltration of Helios^−^ Tregs could aggravate the progression of pneumonia and poor graft function in patients with KTx. Our study is the first to demonstrate that the frequency and Ab No. of Helios^+^ Tregs are reduced in SP patients after KTx, which was tightly associated with disease progression.

CD39^+^ Tregs demonstrated a stronger suppressive ability than CD39^−^ Tregs. In our study, increased CD39^+^ Tregs were observed in patients with SP after KTx, and the expression of CD39^+^ Tregs was positively correlated to creatinine level. We showed that the high frequency of CD39^+^ Tregs indicated a poor prognosis in pneumonia patients post KTx. A previous study has been proved that an increased CD39^+^ Tregs in circulation was closely associated in patients with *mycobacterial* infection [44]. In addition, Huang et al. demonstrated that the level of circulating CD39^+^ Tregs was significantly increased in sepsis patients, and a growing expression of CD39^+^ Tregs was relevant to an unfavorable prognosis for sepsis patients [45]. The progression of infection significantly upregulated the frequency of CD39^+^ Tregs in the whole blood of patients. Our research demonstrated that the frequency of CD39^+^ Tregs was increased in SP patients after KTx, which was tightly associated with pneumonia progression.

Based on CD45RA expression, Tregs can be divided into two different subpopulations: (i) CD45RA^−^ Treg (memory Treg (mTreg)) and (ii) CD45RA^+^ Treg (resting Treg (rTreg)). A previous study demonstrated that mTregs were more suppressive than rTregs and were crucial for the maintenance of immune homeostasis. Meanwhile, increased mTregs are correlated with better transplant outcomes. Previous studies have demonstrated that reduced CD45RA^−^ Tregs are correlated with both acute and chronic rejection in KTx recipients [30, 46]. Our study demonstrated a low frequency of CD45RA^−^ Treg in SP after KTx, indicating poor pneumonia outcomes. In addition to the clustering method mentioned above, some researchers thought that CD45RA^−^ Treg could be further divided into CD45RA^−^Helios^+/-^ Treg and CD45RA^−^CD39^+/-^ Treg. We further discussed the association between different CD45RA^−^ Treg subpopulations and the severity of pneumonia post renal transplantation. We found that the percentage and counts of CD45RA^−^Helios^+^ and CD45RA^−^CD39^−^ Tregs in the SP group were significantly lower than those in the MP group. However, the percentage and counts of CD45RA^−^Helios^−^ and CD45RA^−^CD39^+^ Tregs in the SP group were significantly higher than those in the MP group. Detailed information was presented in supplementary Table 1 and Figure 1. We also analyzed the correlation between different CD45RA^−^ Treg subpopulations and clinical information (Supplementary Figure 2A). We found that the percentage and counts of CD45RA^−^Helios^+^ and CD45RA^−^CD39^+^ Treg were significantly negatively correlated with the level of peripheral BUN and Cr. However, the percentage and counts of CD45RA^−^Helios^+^ and CD45RA^−^CD39^+^ Tregs were significantly positively correlated with the level of peripheral BUN and Cr. Next, we analyzed the correlation between different Treg subpopulations and regular immune status (Supplementary Figure 2B). We discovered that the MFI of CD64 on neutrophils was negatively correlated with the percentage and counts of CD45RA^−^Helios^+^ Treg but positively correlated with the percentage and counts of CD45RA^−^Helios^−^ Treg. Meanwhile, the counts of NK cells were negatively correlated with the percentage and counts of CD45RA^−^Helios^−^ Treg, yet positively correlated with the percentage and counts of CD45RA^−^Helios^+^ Treg. We analyzed the optimal cut-off values by ROC analysis, and the ROC curves were presented in Supplementary Figure 3. All the indices displayed a high AUC. All AUC and cut-off values were displayed in Supplementary Table 2. In Supplementary Table 3, the univariate logistic analysis suggested that the percentage and counts of CD45RA^−^Helios^+^, CD45RA^−^Helios^−^, CD45RA^−^CD39^+^, and CD45RA^−^CD39^−^ Tregs were drastically associated with disease progression. Therefore, indices of CD45RA^−^CD39^+/-^ and CD45RA^−^Helios^+/-^ Tregs showed good potential for the disease progression of pneumonia patients post KTx.

In the current study, we innovatively studied the distribution of different Treg subpopulations in whole blood of SP and MP patients post renal transplantation. A previous study showed that increased Helios^+^ Treg, CD39^+^ Treg, and CD45RA^−^ Tregs were indicated a low rate of transplant rejection in different graft trials [16, 22, 29, 30]. In our research, increased Helios^+^ Treg, decreased CD39^+^ Tregs, and increased CD45RA^−^ Tregs were observed in the MP group. Different from a previous study, our patients were in a state of pneumonia. In the next research, we will further study the relationship between different Treg subpopulations and rejection under a pneumonia state. Through ROC analysis, we found that the AUCs of all indicators were higher than 0.7. The AUC of the percentage of Helios^+/-^ Treg was even higher than 0.85. We thought that Helios^+/-^ Treg, CD39^+/-^ Treg, and CD45RA^+/-^ Treg could become the monitoring indicators for pneumonia patients after transplantation.

Finally, Tregs are known to engage in crosstalk with different immune cells, such as NK cells, monocytes, neutrophils, B cells, and other T cells. For example, NK cell counts are positively correlated with Helios^+^ Tregs in transplant patients [47]. Furthermore, growing evidence indicates that NK cells can modulate Treg responses in infections and inflammatory diseases [48]. Tregs can also suppress conventional T cells and B cells through cytokine secretion and cell-cell contact [49]. In our study, a close correlation was observed between different Treg subpopulations and regular immune cells. The MFI of HLA-DR on monocytes, and CD64 on neutrophils, is a common index to measure the infection of patients. As previous studies demonstrated that the expression of HLA-DR reflected the antigen presentation capacity of monocytes, low expression of HLA-DR indicates a poor prognosis in some infectious diseases like sepsis and virus B hepatitis [50, 51]. In our research, we found that the MFI of HLA-DR on monocytes was higher in the MP group. Our results were consistent with previous studies. Low expression of HLA-DR in monocytes symbolizes the poor prognosis of pneumonia after transplantation. In resting neutrophils, the levels of CD64 are very low. However, it is significantly increased in response to the infection in a very short time [52]. In our study, we found that high expression of CD64 had significantly negative correlation with part of Tregs which play an anti-inflammatory role in pneumonia. High CD64 expression means poor prognosis in infected patients, and this view has also been confirmed in our study.

To obtain a full picture of the immune status for guiding clinical treatment, multiple immune cell subsets need to be detailed. In our previous study, profiles of B cell and T cell subsets were detailed in immune-stable renal allograft recipients [34, 53]. Moreover, we applied machine learning models to determine the connection between immune monitoring and pneumonia in KTx patients, which can contribute to better-individualized therapy [4]. In this previous study, nonetheless, only a regular immune status panel (including TBNK panel, as well as HLA-DR, CD64) was used, and we paid more attention to the comparison between pneumonia and noninfected allograft renal recipients. In our current study, we mainly investigated the association between Treg subsets and the severity of pneumonia patients post kidney transplantation. Compared to the regular immune status panel, Treg subpopulations were also very important for the diagnosis and prognosis of pneumonia patients post kidney transplantation.

Additionally, our Treg panel was the commercial dry powder kit from Beckman Coulter, and each fluorochrome-conjugated antibody or channel was designed and fixed. And the protocol and flow strategy were all solid and fixed by Beckman Coulter. Every center using this commercial panel will do the same steps, not only the experimental protocol but the analyzing flow. For example, Holl et al. used this Treg panel to examine the tumor cellular immunome in cancer patients [54]. Therefore, this panel was designed to uniform the steps among different centers. Of course, in transplantation centers, this panel needs to be broadly applied. We will work to promote the One Study program in transplantation centers. Additionally, it is correct to figure out the association between Treg subsets and infection post KTx was very important and useful for subsequent tolerance induction.

There were some limitations to this study. The morbidity of pneumonia after transplantation is not high because of the gradual improvement of our diagnostic and therapeutic level, so our center collected only 40 valid samples in two years. Our study merely had a comparatively minor number of samples from a single center, which may have contributed to the bias of the data analysis. Meanwhile, our study failed to demonstrate the predicted efficacy for prognosis. Therefore, future large-scale, multicenter, controlled clinical studies are required to fully identify the mechanisms of Treg subpopulations in transplant infection and immunity. Secondly, due to the limitation of detection conditions, we failed to obtain the types of patient's pathogenic bacteria. Whether the distribution of different subpopulations of Tregs is affected by the types of pathogenic bacteria still needs further investigation. Thirdly, patients usually come to see a doctor after showing symptoms of pneumonia. Therefore, we did not get data on the patients' transplanted renal function prior to the onset of infection. We will timely supplement the research on this part in the future.

## 5. Conclusion

This study successfully detailed the distribution of Treg subsets and demonstrated that Treg subpopulations were closely associated with severe pneumonia progression after KTx. The frequencies of total and Helios^−^ Tregs were independent risk factors for SP progression after KTx. Based on the monitoring of Treg subpopulations, better-individualized prevention and therapy might be achieved for patients with SP after KTx.

## Figures and Tables

**Table 1:** Overview of the Treg subset panel dedicated to a specific cell type which is indicated by individual colors.

Excitation (nm)	Blue: 488				Red: 633		Violet: 405
Emission peak (nm)	523	575	692	760	665	783	455
Fluorochrome	FITC	PE	PC5.5	PC7	A647	APC-A750	PB
Treg subset panel	CD45RA	CD25	CD39	CD4	FoxP3	CD3	Helios
Compensation control	CD4	CD4	CD39	CD4	FoxP3	CD3	CD4

FITC: fluorescein isothiocyanate; PE: phycoerythrin; PC5.5/7: phycoerythrin cyanine 5.5/7; A647: Alexa Fluor 647; APC-A750: allophycocyanin Alexa Fluor 750; PB: Pacific Blue.

**Table 2:** Clinical characteristics of the patients.

Characteristics	All (*n* = 40)	Mild (*n* = 27)	Severe (*n* = 13)	*P*
Age (years), mean ± SD	43.93 ± 2.09	45.04 ± 2.56	41.62 ± 3.67	0.4492
Male, *n* (%)	22 (55.00%)	15 (55.56%)	7 (53.85%)	0.99⁣^∗^
BMI (kg/m^2^)	20.32 ± 0.47	20.53 ± 0.56	19.9 ± 0.88	0.5314
Donor, *n* (%)				0.1540⁣^∗^
DCD	35 (87.50)	22 (81.48)	13 (100.00)	
Relative	5 (12.50)	5 (18.52)	0 (0.00)	
Time since transplantation (months), median ± IQR	8.07 ± 22.45	10.93 ± 25.08	7.20 ± 5.50	0.1140^#^
CNI, *n* (%)				0.99⁣^∗^
FK506	38 (95.00)	26 (96.30)	12 (92.31)	
CSA	2 (5.00)	1 (3.70)	1 (7.69)	
Survival, *n* (%)	36 (90.00)	27 (100)	9 (69.23)	0.0078⁣^∗^
Hospital stay period (days), median ± IQR	16 ± 17.50	13 ± 13.50	34 ± 25	0.0078^#^
WBC (10^9^/L), median ± IQR	7.52 ± 4.45	7.69 ± 3.37	6.95 ± 6.57	0.2865^#^
PLT (10^9^/L), median ± IQR	201 ± 96.25	202 ± 80.5	198 ± 138	0.6430^#^
BUN (mmol/L), median ± IQR	9.21 ± 10.81	7.12 ± 5.79	17.26 ± 8.76	0.0002^#^
Cr admission (*μ*mol/L), median ± IQR	123 ± 68.25	119 ± 63	155 ± 255	0.0242^#^
CRP (mg/L), median ± IQR	37.45 ± 54.59	36.72 ± 53.04	41.13 ± 97.08	0.3423^#^
Procalcitonin (ng/mL), median ± IQR	0.10 ± 0.33	0.06 ± 0.16	0.24 ± 7.34	0.0030^#^
Albumin (g/L)	37.0 ± 6.23	39.3 ± 5.46	32.38 ± 5.09	0.0005
Ca^+^ (mmol/L), median ± IQR	2.40 ± 0.24	2.45 ± 0.13	2.26 ± 0.33	0.1324^#^

SD: standard deviation; IQR: interquartile range; DCD: donation after citizens' death; CNI: calcineurin inhibitor; CsA: cyclosporine A; BUN: blood urea nitrogen; Cr: serum creatinine; CRP: C reactive protein; ⁣^∗^tested by Fisher's exact test; ^#^tested by Mann-Whitney *U* test.

**Table 3:** Regular immune monitoring panel of the patients.

Parameters	All (*n* = 30)	MP (*n* = 19)	SP (*n* = 11)	*P*
Monocyte HLA-DR, MFI ± SD	1335 ± 205.0	1671.00 ± 285.80	783.60 ± 184.60	0.0329
Neutrophil CD64, MFI ± SD	595.4 ± 86.70	522.10 ± 112.20	715.5 ± 134.80	0.2872
CD3^+^ T cells/TBNK, mean ± SD (%)	77.72 ± 1.82	76.09 ± 2.41	80.38 ± 2.65	0.2583
CD3^+^ T cells, mean ± SD (/*μ*L)	707.38 ± 77.05	819.2 ± 96.83	524.5 ± 111.1	0.0621
CD8^+^ T cells/TBNK, mean ± SD (%)	34.12 ± 1.80	32.45 ± 1.79	37.06 ± 3.69	0.2207
CD8^+^ T cells, median ± IQR (/*μ*L)	313 ± 245	335.50 ± 204.75	189 ± 241.50	0.2038^#^
CD4^+^ T cells/TBNK, mean ± SD (%)	39.75 ± 1.87	41.60 ± 2.60	36.73 ± 2.33	0.2111
CD4^+^ T cells, mean ± SD (/*μ*L)	367.14 ± 44.49	451.4 ± 58.41	229.2 ± 45.33	0.0125
NK cells/TBNK, mean ± SD (%)	11.23 ± 1.44	12.99 ± 1.90	8.475 ± 1.98	0.1301
NK cells, median ± IQR (/*μ*L)	80 ± 112	110.50 ± 125.75	45 ± 53.50	0.0249^#^
B cells/TBNK, median ± IQR (%)	7.78 ± 8.84	7.03 ± 10.38	8.46 ± 6.20	0.5801^#^
B cells, median ± IQR (/*μ*L)	77 ± 102	93 ± 92.25	41 ± 112	0.5724^#^
CD4/CD8 ratio, median ± IQR	1.11 ± 0.83	1.45 ± 1.01	0.99 ± 0.29	0.2906^#^

^#^Tested by the Mann-Whitney *U* test; others were tested by the unpaired *t*-test. HLA-DR: human leukocyte antigen-DR; MFI: median fluorescence intensity; SD: standard deviation; IQR: interquartile range; TBNK: T, B, and NK cells; NK cells: natural killer cells.

**Table 4:** Treg subset panel of the patients.

Parameters	All (*n* = 40)	Mild (*n* = 27)	Severe (*n* = 13)	*P*
CD4^+^CD25^+^ T cells, median ± IQR (%)	16.43 ± 11.43	17.54 ± 15.93	15.68 ± 8.84	0.3166^#^
CD4^+^CD25^+^ T cells, median ± IQR (/*μ*L)	40.09 ± 56.26	40.48 ± 97.95	32.56 ± 34.53	0.0386^#^
Total Treg, mean ± SD (%)	18.73 ± 1.31	15.26 ± 0.97	25.94 ± 2.56	<0.0001
Total Treg, median ± IQR (/*μ*L)	7.25 ± 11.82	7.21 ± 12.37	7.73 ± 8.27	0.4933^#^
Helios^+^ Treg, mean ± SD (%)	53.77 ± 4.51	65.09 ± 4.43	30.25 ± 6.86	<0.0001
Helios^+^ Treg, median ± IQR (/*μ*L)	3.51 ± 9.38	4.74 ± 9.93	1.07 ± 3.16	0.0127^#^
Helios^−^ Treg, mean ± SD (%)	45.22 ± 4.53	33.57 ± 4.47	69.42 ± 6.58	<0.0001
Helios^−^ Treg, median ± IQR (/*μ*L)	2.78 ± 4.48	2.18 ± 2.64	4.73 ± 4.54	0.0308^#^
CD39^+^ Treg, mean ± SD (%)	32.01 ± 3.26	26.74 ± 3.58	42.95 ± 5.81	0.0177
CD39^+^ T cell, median ± IQR (/*μ*L)	1.68 ± 3.88	1.32 ± 3.20	1.79 ± 3.81	0.4133^#^
CD39^−^ Treg, mean ± SD (%)	66.61 ± 3.17	71.38 ± 3.48	56.69 ± 5.81	0.0278
CD39^−^ Treg, median ± IQR (/*μ*L)	5.40 ± 7.56	6.43 ± 9.44	3.09 ± 5.12	0.1130^#^
CD45RA^+^ Treg, median ± IQR (%)	13.98 ± 15.33	11.63 ± 8.09	27.27 ± 16.14	0.0028^#^
CD45RA^+^ Treg, median ± IQR (/*μ*L)	1.33 ± 1.42	1.17 ± 1.13	1.50 ± 1.70	0.2094^#^
CD45RA^−^ Treg, median ± IQR (%)	85.37 ± 15.35	88.37 ± 8.16	72.73 ± 15.81	0.0018^#^
CD45RA^−^ Treg, median ± IQR (/*μ*L)	5.55 ± 10.63	5.55 ± 10.93	5.33 ± 5.42	0.2639^#^

^#^Tested by the Mann-Whitney *U* test; others were tested by the unpaired *t*-test; SD: standard deviation; IQR: interquartile range.

**Table 5:** Areas under the curve (AUC) and cut-off values of Treg subsets.

Test result variable(s)	Area	SEM	Asymptotic 95% confidence		Cut-off value	Sensitivity%	Specificity%
			Lower bound	Upper bound			
CD4^+^CD25^+^ T cells (/*μ*L)	0.70	0.09	0.53	0.88	<15.62	38.46	100.00
Total Treg (%)	0.84	0.08	0.68	1.00	>19.87	84.62	81.48
Helios^+^ Treg (%)	0.86	0.07	0.73	0.99	<56.91	92.31	81.48
Helios^+^ Treg (/*μ*L)	0.74	0.09	0.57	0.92	<1.740	61.54	85.19
Helios^−^ Treg (%)	0.86	0.07	0.73	0.99	>42.06	92.31	81.48
Helios^−^ Treg (/*μ*L)	0.71	0.09	0.54	0.89	>2.835	76.92	66.67
CD39^+^ Treg (%)	0.72	0.09	0.54	0.89	>34.68	84.62	70.37
CD39^−^ Treg (%)	0.70	0.09	0.52	0.87	<67.84	84.62	62.96
CD45RA^+^ Treg (%)	0.79	0.07	0.65	0.93	>12.37	92.31	55.56
CD45RA^−^ Treg (%)	0.80	0.07	0.66	0.94	<87.15	100.00	51.85

SEM: standard error of mean.

**Table 6:** Risk factors of clinical information for progression by logistic regression.

Variable	Univariate analysis	*P*	Multivariate analysis	*P*
	OR (95% CI)		OR (95% CI)	
Procalcitonin (ng/mL)	1.07 (0.98, 1.17)	0.11		
Albumin (g/L)	0.79 (0.67, 0.93)	0.0044⁣^∗∗^		

BUN (mmol/L)	1.24 (1.07, 1.44)	0.0046⁣^∗∗^	1.24 (1.05, 1.47)	0.01⁣^∗^
Cr admission (*μ*mol/L)	1.01 (1.00, 1.02)	0.0373⁣^∗^		
Hospital stay period (days)	1.10 (1.03, 1.18)	0.0078⁣^∗∗^	1.09 (1.10, 1.18)	0.03⁣^∗^

OR: odds ratio; ⁣^∗^<0.05, ⁣^∗∗^<0.01, and ⁣^∗∗∗^<0.001.

**Table 7:** Risk factors of Treg subpopulations for progression by logistic regression.

Variable	Univariate analysis	*P*	Multivariate analysis	*P*
	OR (95% CI)		OR (95% CI)	
CD4^+^CD25^+^ T cells (/*μ*L)	0.98 (0.95, 1.00)	0.08		
Total Treg (%)	1.24 (1.08, 1.43)	0.003⁣^∗∗^	1.24 (1.06, 1.45)	0.009⁣^∗∗^
Helios^+^ Treg (%)	0.95 (0.92, 0.98)	0.0015⁣^∗∗^		
Helios^+^ Treg (/*μ*L)	0.90 (0.79, 1.03)	0.13		
Helios^−^ Treg (%)	1.06 (1.02, 1.09)	0.0012⁣^∗∗^	1.06 (1.01, 1.10)	0.008⁣^∗∗^
Helios^−^ Treg (/*μ*L)	1.01 (0.94, 1.09)	0.77		
CD39^+^ Treg (%)	1.04 (1.00, 1.09)	0.03⁣^∗^		
CD39^−^ Treg (%)	0.96 (0.92, 1.00)	0.04⁣^∗^		
CD45RA^+^ Treg (%)	1.07 (1.00, 1.15)	0.04⁣^∗^		
CD45RA^−^ Treg (%)	0.93 (0.85, 0.99)	0.03⁣^∗^		

OR: odds ratio; ⁣^∗^<0.05, ⁣^∗∗^<0.01, and ⁣^∗∗∗^<0.001.

## Data Availability

The raw data supporting the conclusions of this article will be made available by the authors upon request, without undue reservation.

## Supplementary Materials

Supplementary Materials Supplementary Table 1: CD45RA^−^ Treg subset panel of the patients. Supplementary Table 2: areas under the curve (AUC) and cut-off values of C45RA^−^ Treg subsets. Supplementary Table 3: risk factors of Treg subpopulations for progression by logistic regression. Supplementary Figure 1: the distribution and Ab No. of CD45RA^−^ Treg subpopulations in MP and SP patients. Supplementary Figure 2: correlation analysis of CD45RA^−^ Treg subpopulations with clinical information and regular immune status. (A) The heatmap of the correlations between different CD45RA^−^ Treg subpopulations and the clinical information of the patients. (B) The heatmap of the correlations between different CD45RA^−^ Treg subpopulations and the regular immune indexes of the patients. BUN: blood urea nitrogen; Cr: serum creatinine. Red indicates positive correlations while blue indicates negative ones. ∗∗∗ means *P* < 0.01, ∗∗ means *P* < 0.05, and ∗ means *P* < 0.1. Supplementary Figure 3: ROC curve of CD45RA^−^ Treg subpopulations in MP and SP patients. (A) ROC curve of CD45RA^−^Helios^+^ Treg (% and Ab No.). (B) ROC curve of CD45RA^−^Helios^−^ Treg (% and Ab No.). (C) ROC curve of CD45RA^−^CD39^+^ Treg (% and Ab No.). (D) ROC curve of CD45RA^−^CD39^−^ Treg (% and Ab No).
