# Supplementary material for: The Association between Regulatory T Cell Subpopulations and Severe Pneumonia Post Renal Transplantation
Source: J Immunol Res. 2022 Apr 9;2022:8720438. doi: 10.1155/2022/8720438 (PMC9013297; doi:10.1155/2022/8720438)
Supplement: Supplementary Materials — Supplementary Table 1: CD45RA− Treg subset panel of the patients. Supplementary Table 2: areas under the curve (AUC) and cut-off values of C45RA− Treg subsets. Supplementary Table 3: risk factors of Treg subpopulations for progression by logistic regression. Supplementary Figure 1: the distribution and Ab No. of CD45RA− Treg subpopulations in MP and SP patients. Supplementary Figure 2: correlation analysis of CD45RA− Treg subpopulations with clinical information and regular immune status. (A) The heatmap of the correlations between different CD45RA− Treg subpopulations and the clinical information of the patients. (B) The heatmap of the correlations between different CD45RA− Treg subpopulations and the regular immune indexes of the patients. BUN: blood urea nitrogen; Cr: serum creatinine. Red indicates positive correlations while blue indicates negative ones. ∗∗∗ means P < 0.01, ∗∗ means P < 0.05, and ∗ means P < 0.1. Supplementary Figure 3: ROC curve of CD45RA− Treg subpopulations in MP and SP patients. (A) ROC curve of CD45RA−Helios+ Treg (% and Ab No.). (B) ROC curve of CD45RA−Helios− Treg (% and Ab No.). (C) ROC curve of CD45RA−CD39+ Treg (% and Ab No.). (D) ROC curve of CD45RA−CD39− Treg (% and Ab No). [file 8720438.f1.docx]

**Supplementary materials:**

**Supplementary Table 1. CD45RA^-^Treg subset panel of the patients**

| **Parameters** | **All (n=40)** | **Mild (n=27)** | **Severe (n=13)** | **P** |
| --- | --- | --- | --- | --- |
| CD45RA^-^Helios^+^Treg, mean ± SD (%) | 64.27±25.58 | 76.54±18.03 | 38.79±19.49 | <0.0001 |
| CD45RA^-^Helios^+^Treg, mean ± SD (/μl) | 53.10±23.62 | 64.65±17.42 | 29.12±15.27 | <0.0001 |
| CD45RA^-^Helios^-^Treg, mean ± SD (%) | 34.63±25.82 | 22.14±17.78 | 60.56±20.05 | <0.0001 |
| CD45RA^-^Helios^-^Treg, mean ± SD (/μl) | 27.34±18.78 | 18.88±14.11 | 44.91±14.81 | <0.0001 |
| CD45RA^-^CD39^+^Treg, mean ± SD (%) | 24.45±19.68 | 28.09±16.74 | 47.68±19.25 | 0.0021 |
| CD45RA^-^CD39^+^Treg, mean ± SD (/μl) | 27.81±16.26 | 23.91±15.25 | 35.93±15.80 | 0.0186 |
| CD45RA^-^CD39^+^Treg, mean ± SD (%) | 65.39±19.99 | 72.24±16.68 | 51.14±19.25 | 0.001 |
| CD45RA^-^CD39^+^Treg, mean ± SD (/μl) | 53.37±18.77 | 60.89±15.91 | 37.76±14.33 | 0.0002 |

^#^Tested by Mann-Whitney U test, others were tested by unpaired t test.

**Supplementary Table 2. Areas under the curve (AUC) and cut-off values of C45RA^-^Treg subsets**

| **Test result variable(s)** | **Area** | **SEM** | **Asymptotic 95% confidence** | | **Cut-off value** | **Sensitivity%** | **Specificity%** |
| --- | --- | --- | --- | --- | --- | --- | --- |
|  |  |  | **Lower bound** | **Upper bound** |  |  |  |
| CD45RA^-^Helios^+^ Treg (%) | 0.92 | 0.04 | 0.83 | 1.00 | < 70.25 | 100.00 | 74.07 |
| CD45RA^-^Helios^+^ Treg (/μl) | 0.88 | 0.06 | 0.78 | 0.99 | <48.14 | 85.71 | 84.62 |
| CD45RA^-^Helios^-^ Treg (%) | 0.92 | 0.04 | 0.84 | 1.00 | > 30.63 | 100.00 | 77.78 |
| CD45RA^-^Helios^-^ Treg (/μl) | 0.89 | 0.05 | 0.79 | 0.99 | > 19.50 | 100.00 | 65.38 |
| CD45RA^-^CD39^+^ Treg (%) | 0.79 | 0.08 | 0.62 | 0.95 | > 42.16 | 76.92 | 81.48 |
| CD45RA^-^CD39^+^ Treg (/μl) | 0.74 | 0.08 | 0.57 | 0.90 | > 30.61 | 71.43 | 73.08 |
| CD45RA^-^CD39^-^ Treg (%) | 0.90 | 0.08 | 0.64 | 0.96 | < 58.08 | 76.92 | 81.48 |
| CD45RA^-^CD39^-^ Treg (/μl) | 0.84 | 0.07 | 0.71 | 0.97 | < 42.25 | 71.43 | 92.31 |

SEM: standard error of mean.

**Supplementary Table 3. Risk factors of Treg subpopulations for progression by logistic regression**

| **Variable** | **Univariate analysis** |  |
| --- | --- | --- |
|  | **OR (95%CI)** | **P** |
| CD45RA^-^Helios^+^ Treg (%) | 0.92(0.86,0.96) | 0.0009** |
| CD45RA^-^Helios^+^ Treg (/μl) | 0.90(0.84,0.95) | 0.0009** |
| CD45RA^-^Helios^-^ Treg (%) | 1.09(1.05,1.16) | 0.0008** |
| CD45RA^-^Helios^-^ Treg (/μl) | 1.11(1.05,1.19) | 0.0009** |
| CD45RA^-^CD39^+^ Treg (%) | 1.06(1.02,1.12) | 0.0075** |
| CD45RA^-^CD39^+^ Treg (/μl) | 1.05(1.01,1.10) | 0.0354* |
| CD45RA^-^CD39^-^ Treg (%) | 0.94(0.89,0.98) | 0.0052** |
| CD45RA^-^CD39^-^ Treg (/μl) | 0.92(0.86,0.96) | 0.0017** |

OR: odds ratio, *<0.05, **<0.01.


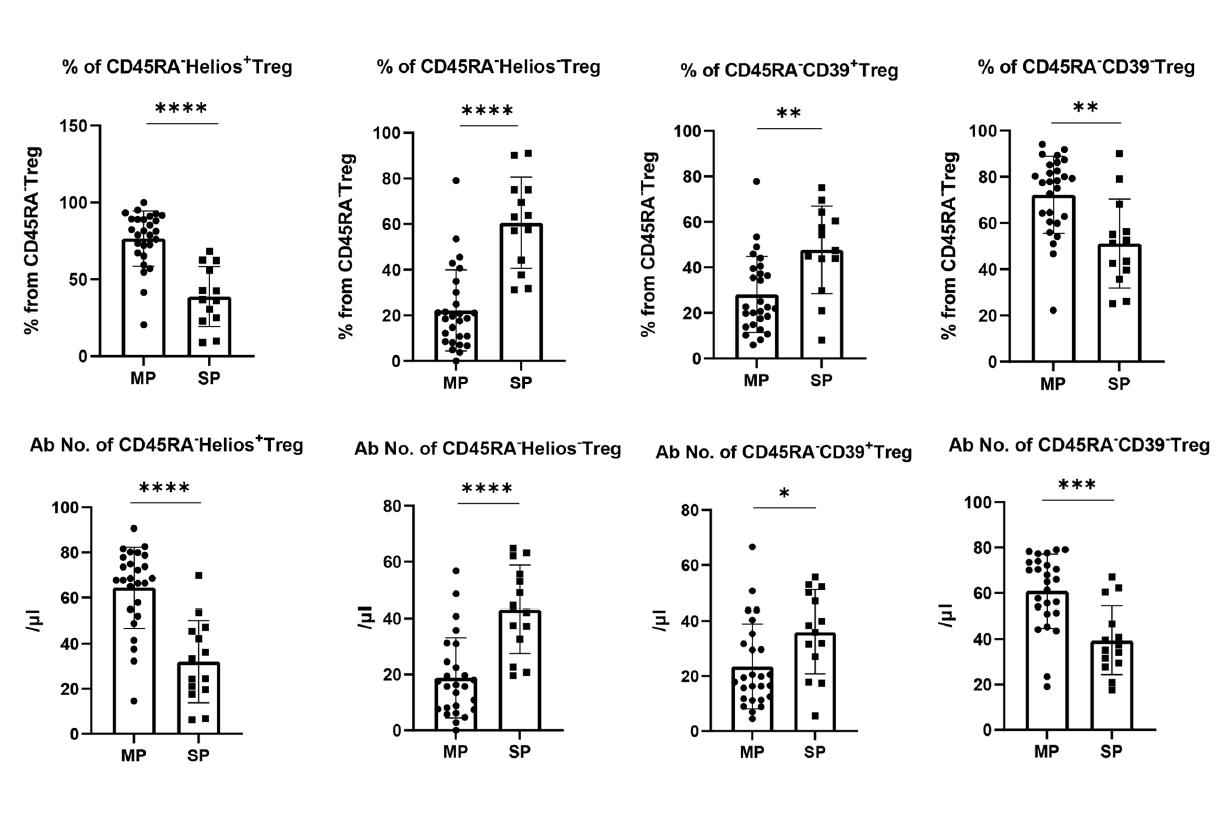


**Supplementary Figure 1. The distribution and Ab No. of CD45RA^-^Treg subpopulations in MP and SP patients.**

**
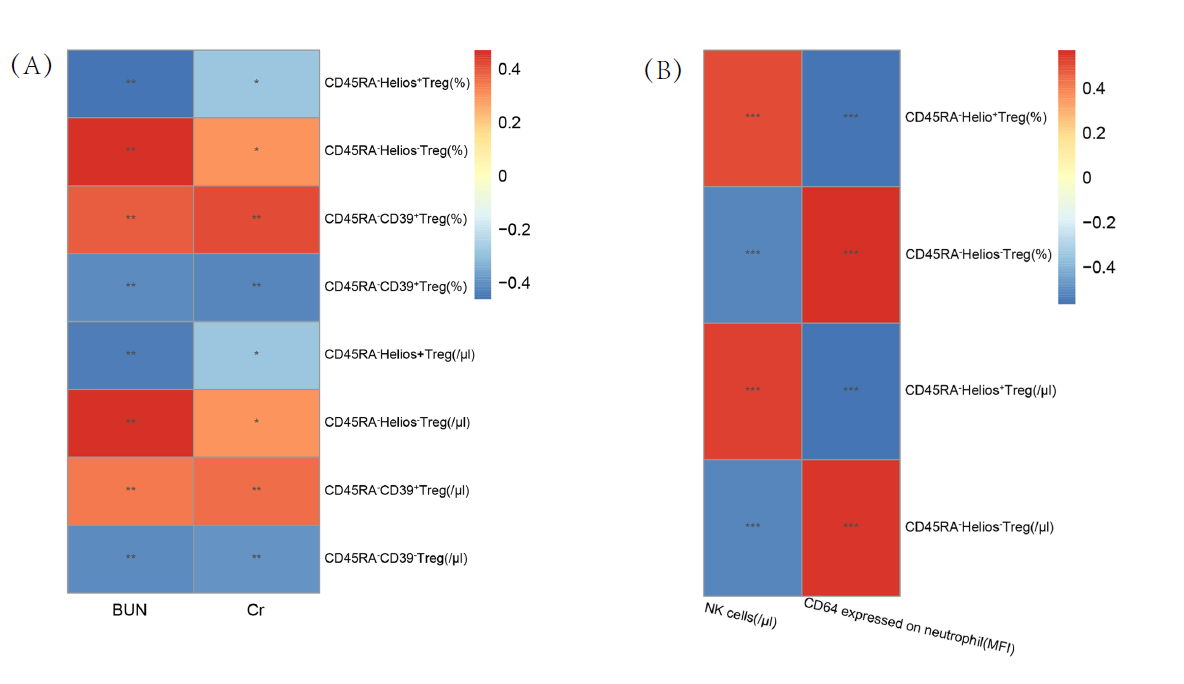
**

**Supplementary Figure 2. Correlation analysis of CD45RA^-^Treg subpopulations with clinical information and regular immune status**. A. The heatmap of the correlations between different CD45RA^-^Treg subpopulations and the clinical information of the patients. B. The heatmap of the correlations between different CD45RA^-^Treg subpopulations and the regular immune indexes of the patients. BUN: ﻿blood urea nitrogen, Cr: serum creatinine. Red indicates positive correlations while blue indicates negative ones.

**
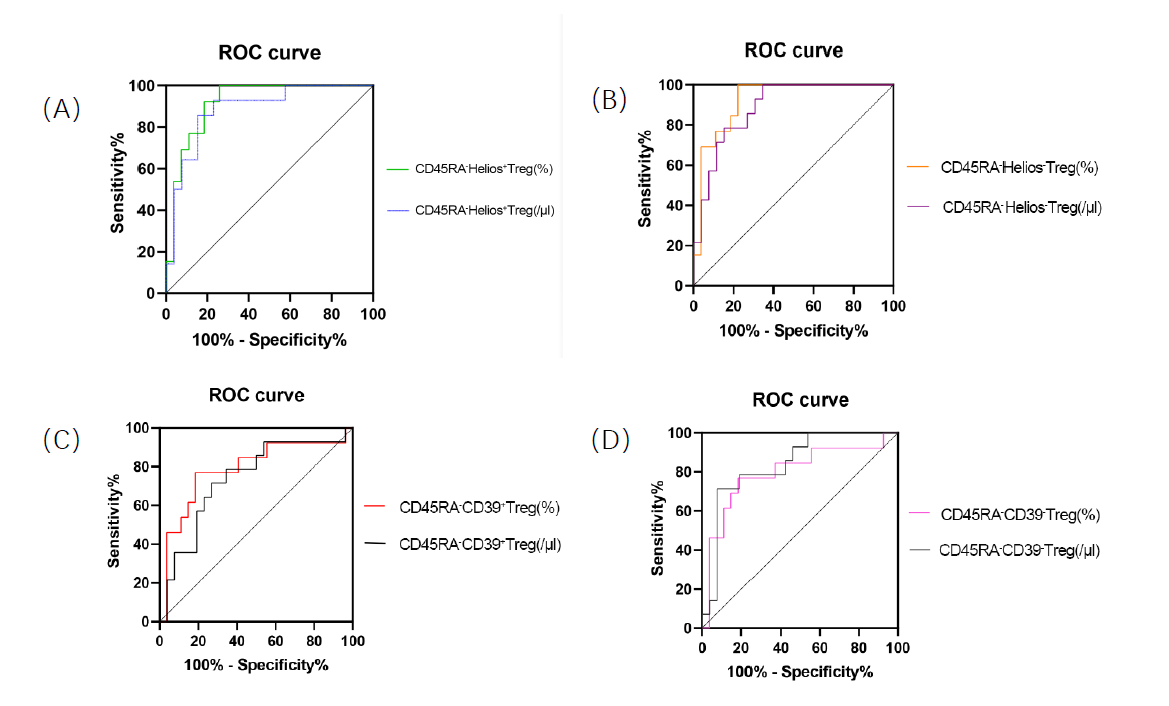
**

**Supplementary Figure 3. ROC curve of CD45RA^-^Treg subpopulations in MP and SP patients.** A. ROC curve of CD45RA^-^Helios^+^Treg (% and Ab No.). B. ROC curve of CD45RA^-^Helios^-^Treg (% and Ab No.). C. ROC curve of CD45RA^-^CD39^+^Treg (% and Ab No.). D. ROC curve of CD45RA^-^CD39^-^Treg (% and Ab No).
